# Epidemiology of coronavirus disease 2019 (COVD-19) outbreak cases in Oyo State South West Nigeria, March-April 2020

**DOI:** 10.11604/pamj.supp.2020.35.2.23832

**Published:** 2020-06-23

**Authors:** Aishat Bukola Usman, Olubunmi Ayinde, Akinfemi Akinyode, Abass Gbolahan, Bashir Bello

**Affiliations:** 1 African Field Epidemiology Network, Abuja, Nigeria; 2 Nigerian Field Epidemiology and Laboratory Training Program, Nigeria; 3 Department of Public Health, Oyo State Ministry of Health, Ibadan Nigeria; 4 Department of Planning, Research and Statistics, Oyo State Ministry of Health, Ibadan Nigeria

**Keywords:** COVID-19, epidemiology, outbreak, Oyo State

## Abstract

**Introduction:**

On March 17th, 2020, Oyo State recorded her first case of COVID-19 through a United Kingdom returnee. Oyo State Ministry of Health with the support of technical and development partners responded quickly and effectively to contain the outbreak. The outbreak was characterized by place, person and time.

**Methods:**

Field investigations were conducted and contact tracing and follow up done, all confirmed cases were identified, line-listed and analyzed using Epi-info version 7.

**Results:**

A total of 34 confirmed cases were identified all within the capital city of Oyo State and two transferred from other states. The mean age was 49.1 ± 2.0 years with over 40% within the age group 50-59 years. There were 11(35.5%) health care workers infection. The case-fatality was 6.5%. The epidemic curve initially shows a typical propagated pattern, followed by a point source; though atypical.

**Conclusion:**

Outbreak of COVID-19 was confirmed in Oyo State. Field investigation provided information on the characteristics of persons, time and place. Intensified surveillance activities such as contact tracing and follow- up, drive through testing and active case search were useful in early case detection and control of the outbreak.

## Introduction

Public Health activities are presently affected by emerging diseases globally. Socio-economic, environmental and ecological factors are thought to be drivers of this emergence which has also put a lot of pressure on the health systems and global economy [1-3]. Coronavirus disease 2019 (COVID-19) is novel and was first discovered as an outbreak amidst respiratory illness cases in Wuhan City, Hubei Province, China [1]. It can be defined as an illness caused by a novel coronavirus now called severe acute respiratory syndrome coronavirus 2 (SARS-CoV-2; formerly called 2019-nCoV). Human coronavirus is one of the main pathogens of respiratory infection. The two highly pathogenic viruses, SARSCoV and MERSCoV, cause severe respiratory syndrome in humans and four other human corona viruses (HCoVOC43, HCoV229E, HCoVNL63, HCoV-HKU1) induce mild upper respiratory disease. The sequence of SARS-CoV-2 is relatively different from the six other coronavirus subtypes [4]. It was first reported to the WHO on December 31, 2019 and the WHO declared the COVID-19 outbreak a world wide health emergency on January 30, 2020 [2,3]. On March 11, 2020, the WHO declared COVID-19 a global pandemic. The first such designation since declaring H1N1 influenza so in 2009 [5].

The WHO, recently termed the illness caused by SARS-CoV-2 “coronavirus disease 2019” with the acronym COVID-19. In order to avoid stigmatizing the virus’s origins in terms of populations, geography, or animal associations the name COVID-19 was chosen. The International Committee on Taxonomy of Viruses is a group who studied the Coronavirus and issued a statement on February 11, 2020 announcing an official designation for the novel virus: severe acute respiratory syndrome coronavirus 2 (SARS-CoV-2) [6]. Furthermore, the Centre for Disease Control (CDC) classified high risk individuals as those in close contacts of infected persons, travellers arriving from locations where local spread has been reported, persons in areas with ongoing local transmission and healthcare workers caring for patients with COVID-19 [7]. COVID-19 presents in different ways ranged from severe illness and mortality to mild symptoms and even been asymptomatic. Symptoms may develop between 2 to 14 days following exposure to the virus [8]. A pooled analysis of 181 confirmed cases of COVID-19 outside Wuhan, China, found the mean incubation period to be 5.1 days and that 97.5% of individuals who developed symptoms did so within 11.5 days of infection [9]. Wu and McGoogan reported that, among 72,314 COVID-19 cases reported to the Chinese Center for disease Control and Prevention (CCDC), 81% were mild (absent or mild pneumonia), 14% were severe (hypoxia, dyspnea, >50% lung involvement within 24-48 hours), 5% were critical (shock, respiratory failure, multiorgan dysfunction), and 2.3% were fatal [10].

Transmission rates are unknown for SARS-CoV-2; however, there is evidence of human-to-human transmission. Transmission is believed to occur via respiratory droplets (which usually cannot travel for more than 6 feet) from coughing and sneezing, as with other respiratory pathogens, including influenza and rhinovirus [11]. Individuals can be infected by contact with viruses releases in respiratory secretion or thru contact with infected mucus membrane. The virus can also persist on surfaces to varying durations and degrees of infectivity. One study found that SARS-CoV-2 remained detectable for up to 72 hours on some surfaces with decreasing infectivity over time. Notably, the study reported that no viable SARS-CoV-2 was measured after 4 hours on copper or after 24 hours on cardboard [12]. Data have suggested that asymptomatic patients are still able to transmit infection. This raises concerns for the effectiveness of isolation [13,14] Zou et al followed viral infections through nasal and throat swabs in a small cohort of patients. They found increases in viral loads at the time that the patients became symptomatic. However, on day seven after presumed infection a patient was observed to be shedding viruses and yet remained asymptomatic. This paper describes the epidemiology of COVID-19 cases in Oyo State, South West, Nigeria: March-April 2020.

## Methods

**Outbreak setting:** Oyo State llocated in the South-West geopolitical zone of Nigeria with her capital in Ibadan. Oyo State consists of 33 Local Governments Areas (LGAs) and 29 Local Council Development Areas and has a projected population of 8,929,410 with annual growth rate of 3.2 [15]. The land mass of the state is of 28,454 square kilometres and it is bounded in the south by Ogun State, Kwara State in the north, partly bounded by Ogun State and the Republic of Benin in the west, while in the East by Osun State. Ibadan is the capital of Oyo State and Nigeria largest city by geographical area. It has a population of over 3 million, with 11 Local government Areas in its metropolis [15].

**The index case investigation:** a 42-year-old male UK returnee on board with Virgin airline through Muritala Muhammed International Airport on 12th March 2020.He was picked up by his driver and drove straight home to Bodija Ibadan. He immediately went on self-isolation but developed symptoms of fever with two temperature readings of 37.9 and 38.2 on 13th March 2020.Laboratory sample of his nasopharyngeal swab was collected on 16th March 2020 and the result came back positive for COVID-19 on 21st March 2020. The Federal ministry of health was notified of the outbreak.

**Field investigation:** the investigation team comprising Disease and Surveillance Notification officers in the state and LGAs, Nigeria Field Epidemiology and Laboratory Training Programme (NFELTP) residents, volunteers, UNICEF, WHO and Nigeria Centers for Disease control officials. The team working at the COVID-19 Emergency Operation Center(CEOC) planned the administrative, consultative and logistic measures. Visits were made to homes to identify contacts of suspected and confirmed cases or deaths where the outbreak occurred or was rumored to have occurred. As part of active surveillance and containment measures undertaken, contact tracing and follow up measures were adapted from the NCDC guidelines [16].

**Contact and Case definitions:** contact of confirmed case was individuals who are associated with some sphere of activity of the case and may have similar or other exposures as the case. Contacts can include household members, other family contacts, visitors, neighbors, colleagues, teachers, classmates, co-workers, social or health workers, and members of a social group. Close contact: any person who had contact (within 1 meter) with a confirmed case during their symptomatic period, including one day before symptom onset. Social and health care workers contact: any social or health care worker, who provided direct personal or clinical care, or examination of a symptomatic confirmed case of 2019nCoV or within the same indoor space, when an aerosol generating procedure was implemented. Household (or closed setting) contact: Any person who has resided in the same household (or other closed setting) as the primary COVID-19 case. These contacts were identified, listed and subsequently followed up every day for 14 days and observed for symptoms, including development of fever (37.5°C axillary temperature) becoming a case, or otherwise discarded. To identify cases or deaths from COVID-19, we defined a suspected case as any person (including severely ill patients) with any of the following symptoms: fever, cough or difficulty in breathing who within 14 days before the onset of illness had any of the following exposures: history of travel to any high risk country( UK, USA, China, Korea, Iran, Italy) with confirmed and ongoing community transmission of SARS-CoV-2 OR Close contact with a confirmed case of COVID-19 OR Exposure to a healthcare facility where COVID-19 case(s) have been reported. A probable case was defined as a suspect case for whom testing for COVID-19 is inconclusive or for whom testing was positive on a pan-coronavirus assay. Whereas a confirmed case was defined as any person with laboratory confirmation of SARS-CoV-2 infection with or without signs and symptoms by RT-PCR. One single case of COVID-19 was considered an outbreak.

**Contact tracing:** contact listing form and contact follow-up forms were used for listing of contacts and follow up. The contact listing form obtained information on name, age, sex, address, contact, and phone numbers. The contact follow-up form obtained information on name, age, sex, address, date of last contact, type of contact, household information, phone numbers and clinical data of contacts.

**Persons of interest:** manifest of persons who came into Oyo State from high risk countries or states was obtained from the Airlines and point of entry officials. They were followed up for 14 days to rule out symptoms of COVID-19.

**Surveillance activities:** enhanced surveillance activities included accelerated community drive through testing, active case search in health facilities, communities as well as screening of passengers at the points of entry for COVID-19; In the health care facilities, we searched for symptoms of Severe Acute Respiratory infection in the records, HCW illness, sick leave or unexplained absenteeism. For the community, house-house case search was done to identify any person in the household with symptoms of Severe Acute Respiratory Infection. The community drive through testing provided opportunity for high risk contacts to get tested for COVID-19.

**Rumor management:** three toll free lines were made available to the public for call in if there is anyone with symptoms and signs of COVID-19 in the community. The call centre had volunteers who ran three shifts daily, six (6) volunteers ran the morning shift from 8am to 1pm, 6 volunteers during the afternoon shift from 1pm to 6pm and four (4) volunteers during the night shift from 6pm to 8pm. Calls are received by volunteers at the call centre from the public, responses are made based on the algorithm and action were taken as appropriate. The calls were screened for symptoms of COVID-19 and travel history to high risk countries or state.

**Points of entry:** the port health officers conducted screening of travelers at the airport using infrared thermometers as well as provision of hand washing points and sanitizers at the airport. The team also established a Port Sanitary Group (a network of various stakeholders) comprising Nigeria Immigration, Nigeria Customs Service, Nigeria Quarantine Service and the Department of State Service to curb influx of returnees through the international land borders. Attention was also focused on screening passengers on long vehicles bringing in large number of people from the Northern part of the country. These passengers may include returnees that travelled in from countries that share the Northern border with Nigeria. e.g. Niger and Chad.

**Isolation and treatment centers:** patients were managed at the Agbami Isolation Centre, Jericho, Ibadan, infectious disease center at Olodo, isolation unit of University College Hospital and other centers outside Ibadan. These facilities provided 24-hour care with clinical staff including Doctors, Nurses and ancillary staffs trained on COVID-19 case management. These facilities operated with Laboratory support from Lagos State University Teaching Hospital, the Virology Laboratory of Redeemer´s University in Ede Osun State and the University College Hospital Virology laboratory.

**Data analysis:** a line-list of cases was created consisting of data on the date samples were collected, age, sex, occupation, exposure type, date of presentation, presenting symptoms and outcomes was used for descriptive analysis and generation of an epidemic curve. The investigation was a part of public health response and review and was approved by the Oyo State ethics and review committee with approval number AD 13/479.1774b.

## Results

Table 1 shows the characteristics of the cases, in terms of age, sex, exposure types, location and outcomes. The mean age was 42.9 ± 2.0 years with most of the cases within the age group 51-60 years. Among the cases, 74.3% were males, with 58.3% exposures within Nigeria, a little above half (58.3%) of the cases had secondary education or higher and % were by occupation. Almost all the cases recovered and alive (94.4%) with case fatality rate of 5.6%. The outbreak occurred in eight LGAs in the metropolis and two LGAs outside Ibadan.

**Table 1:** Characteristics of COVID-19 cases in Ibadan metropolis February-April 2020

Variables	Frequency(n=36)	Percent
**Age**		
21-30	11	30.6
31-40	3	8.3
41- 50	7	19.4
51- 60	11	30.6
61-70	4	11.1
Mean age: 42.9±2.0		
**Sex**		
Male	26	72.2
Female	10	27.8
**History of travel from high risk countries/states**		
Yes	16	27.8
No	20	72.2
**Educational Level**		
Primary or less	15	41.6
Secondary and above	21	58.3
**Occupation**		
Health care workers	11	30.1
Civil servants	7	19.4
Farmers	6	16.7
Traders/Artisans	12	33.3
**Type of Exposure**		
Outside Nigeria	15	41.7
Within Nigeria	21	58.3
**Place of Residence**		
Akinyele	1	2.7
Ibadan North	18	50.0
Ibadan North West	1	2.7
Ibadan South East	1	2.7
Ibadan South West	2	5.6
Ido	3	8.6
Lagelu	2	5.4
Oluyole	2	5.4
Orire	5	13.9
Surulere	1	2.7
**Outcome**		
Alive	34	94.4
Dead	2	5.6

Others are caterer, fashion designer, student and trader

**Figure 1:**
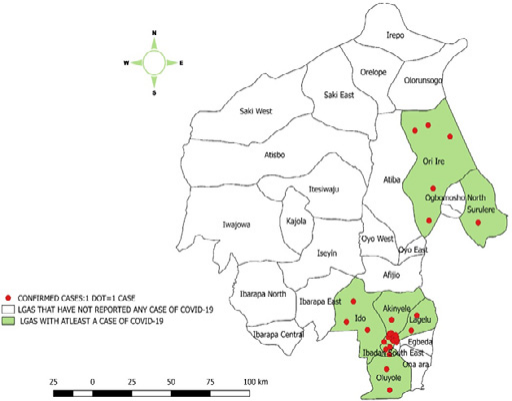
Of Oyo State highlighting the LGAs of confirmed COVID-19 cases

**Epidemiology of the index case:** a 42-year-old male UK returnee on board with Virgin airline through Muritala Muhammed International Airport on 12th March 2020. He was picked up by his driver and drove straight home to Bodija Ibadan. He immediately went to self-isolation but developed symptoms of fever with two temperature readings of 37.9 and 38.2 on 13th March 2020. Laboratory sample of his nasopharyngeal swab was collected on 16th March 2020 and the result came back positive for COVID-19 on 21st March 2020. The Federal Ministry of health was notified of the outbreak. He was moved to Agbami Treatment center for proper case management. His samples were retested twice and became negative. He was discharged on the 30th March 2020 (Figure 1).

**Figure 2:**
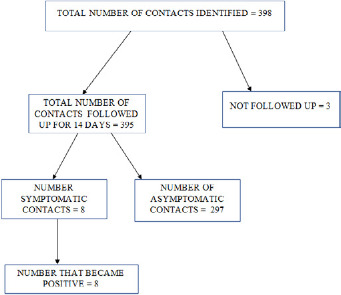
Contacts of COVID-19 cases in Oyo State, March to April 2020

**Outcome of identification of cases and contact tracing:** thirty-four cases were confirmed from the laboratory, two confirmed from Lagos and Kano States were transferred to Oyo State, three hundred and ninety-eight contacts were identified with these cases and 395 were followed up for 14days.Of these, 287 exited 14 days follow up without any symptoms, while 8 were symptomatic and tested positive. The 287 contacts that were asymptomatic were not tested because the National guidelines as at time of this data collection says only symptomatic contacts should be tested [17]. One hundred and thirty-one contacts(39.5%) were from Ibadan North Local Government Area (Figure 2).

**Outcome of persons of interest(POIs):** one hundred and fifty-one POIs were identified. Of these,52(34.4%) became symptomatic and 10(19.2%) of the symptomatic became positive.

**Rumor Management:** a total of 1,552 calls were received,86% were resolved and 14% were escalated to the surveillance team for follow up.

**Clinical features of cases:** half of the cases (55.6%) were asymptomatic as at the time they were confirmed. For the symptomatic cases,61.5% had fever as their first symptom while 38.5% had cough. Only 9.1% of the cases presented with symptoms like chills, difficulty in breathing, fatigue and headache.

**Figure 3:**
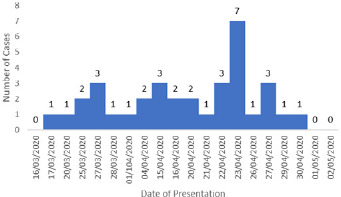
Epi-curve of confirmed cases of COVID-19 in Oyo State March to April 2020

**Epi-Curve:** the epidemic curve (Figure 3) initially shows a typical propagated pattern outbreak, followed by a point source pattern; though atypical. The median incubation period was 12 days and serial interval for the first wave of infection was 4 days.

## Discussion

This is a descriptive epidemiology of COVID-19 cases characterized by persons, time and place between March and April 2020 in Oyo State. One-third of the cases were health care workers who reported been in contact with infected patient as seen in other studies [18]. All health care workers cases reported direct physical contact with an infected person. This is more obvious among the health workers being in the frontline. Other factors may include the lack of consideration given to potential droplets from asymptomatic infected persons which were not overtly visible. The role of direct physical contact in the absence of visible symptoms will have a great implication for the containment of the disease. The case fatality of SARS CoV 2 has been reported to be higher than 10% and MERS-CoV at more than 35% [6,19]. Findings from this review was lower than that of previous studies [20]. Also, the case fatality recorded was low compared to Lagos and FCT [19] and even in the West African region where number of deaths ranges from 1-24 [21]. This pattern of low case fatality was also observed among health workers, who are the high-risk group, this may be suggestive of an effective outbreak response.

Emerging evidence suggests that more men than women are dying, potentially due to sex-based immunological or gendered differences, such as patterns and prevalence of smoking [22]. Another reason may be that men often don’t present in the health-care delivery system until they have greater symptomatology However, we observed a greater number of men than women were affected as observed from data from other states in Nigeria, other African countries and globally, males were the most affected [19,21]. MERSCoV and SARS CoV were also found to infect more males than females [23,24]. The reduced susceptibility of females to viral infections could be attributed to the protection from X chromosome and sex hormones, which play an important role in innate and adaptive immunity [25]. From research sex-disaggregated data for COVID-19 showed equal number of cases between men and women so far, but there seem to be sex differences in mortality and vulnerability to the disease [21]. The host´s immune status is also one of the important factors, old age, obesity, and presence of co-morbidity might be associated with increased mortality as also seen in this study [26]. The two deaths were males, associated with comorbidities and age above 50yrs. This shows that early detection and timely treatment of severe cases is very important.

Fever and cough were the most commonly reported symptoms at presentation. Only very few presented with headache, fatigue, abdominal symptoms and chills. These are consistent with clinical symptoms being reported from the concurrent epidemics in other countries within the sub region [27].

The current outbreak in Oyo State can be said to be small compared to concurrent outbreak in other Nigerian states Lagos, FCT and Kano States [19]. However, in terms of spread, cases were identified in one major urban LGA that is in the heart of Ibadan, Oyo State capital. This is similar to current outbreak reported in Lagos and Kano States and other countries where majority of the cases of COVID-19 were in the major cities although there are cases from rural areas too [28]. The epidemic curve initially shows a typical propagated pattern, followed by a point source; though atypical. This is in tandem with the epidemic curve described in the outbreak in China with a typical point source pattern [29]. The median incubation period we obtained is consistent with the known incubation of COVID-19. There was primary wave of infection from the two of the cases in the current outbreak. The serial interval for the first wave was 4days which is in line with previous studies on COVID-19 [30]. We were not able to explore the data further to generate the reproductive rate due to the small number of cases. This is a preliminary finding on the ongoing outbreak, we recognize the challenges that the field investigation may have imposed on our findings. The teams of public health workers used in the response had no previous experience in response to COVID-19 outbreaks although they were highly skilled health professionals who have participated in the response to Lassa fever and Ebola outbreaks in Nigeria. However, misclassification bias was minimized by using a standard case definition during case identification and contact listing. Data cleaning was performed at every stage of the data collection process. The data in this study is only an early review of the epidemiological characteristics of COVID-19 which may not reflect true outbreak pattern for the state as the outbreak is ongoing. Also, in generating the epi-curve, day of presentation was used as opposed to the day of onset of symptoms because not all cases were symptomatic. This may be a form of limitation.

## Conclusion

This review is in response to the outbreak of COVID-19 in Oyo State, which was of clustering onset, is more likely to infect older men with comorbidities, and can result in severe and even fatal respiratory diseases. However, to ensure containment of COVID-19 outbreak in Oyo State enhanced field investigation, surveillance measures, including contact tracing and follow-up, effective case management and community sensitization efforts must be intensified.

### What is known about this topic

- COVID-19 is a novel virus with pandemic and the mode of transmission is by droplet spread;
- COVID-19 can be prevented by performing hand hygiene, using face mask and maintaining social distancing.

### What this study adds

- This study describes the characteristics of early COVID-19 cases in Oyo State;
- This serves as a contribution to the body of knowledge on COVID-19;
- Useful as a guide in further response activities to the pandemic in the state.

## Competing interests

The authors declare no competing interests.
